# Supplementary material for: Cytosolic TOP3α facilitates mitochondrial DNA sensing by cGAS
Source: EMBO Rep. 2025 Oct 30;26(23):5959–81. doi: 10.1038/s44319-025-00614-2 (PMC12678531; doi:10.1038/s44319-025-00614-2)
Supplement: Supplementary file 1 — Appendix [file 44319_2025_614_MOESM1_ESM.pdf]

Appendix Figures for "Cytosolic TOP3α facilitates mitochondrial DNA sensing by cGAS"

Appendix Figure S1. TOP3α downregulation does not alter mitochondrial membrane potential and ROS level. 2
Appendix Figure S2. Subcellular fractionation does not cause the release of mitochondrial matrix components. 3
Appendix Figure S3. Cytosolic TOP3α promotes DNA-cGAS interaction. 4
Appendix Figure S4. Depleting cytosolic TOP3α impairs mtDNA-cGAS interaction. 5
Appendix Figure S5. Mitochondrial overloaded TOP3α is required to cause mtDNA release. 6
Appendix Figure S6. Enhancing mitochondrial targeting of TOP3α impairs mitochondral membrane potential. 7
Appendix Figure S7. Complete blots of Figure EV4B. 8
Appendix Figure S8. Loading control for Figure 5I. 9
Appendix Figure S9. TOP3α-G250D suppresses the interaction of cGAS with mtDNA. 10
Appendix Figure S10. The IP efficiency of biotinylated DNA. 11
Appendix Figure S11. TOP3α-G250D suppresses the interaction of cGAS with mtDNA. 12
Appendix Figure S12. Independent repeat experiments related to Figure 2. 13
Appendix Figure S13. Independent repeat experiments related to Figure 3. 14
Appendix Figure S14. Independent repeat experiments related to Figure 5. 15
Appendix Figure S15. Independent repeat experiments related to Figure 6. 16
Appendix Figure S16. Independent repeat experiments related to Figure EV3. 17
Appendix Figure S17. Independent repeat experiments related to Figure EV4. 18
Appendix Figure S18. Independent repeat experiments related to Figure EV5. 19

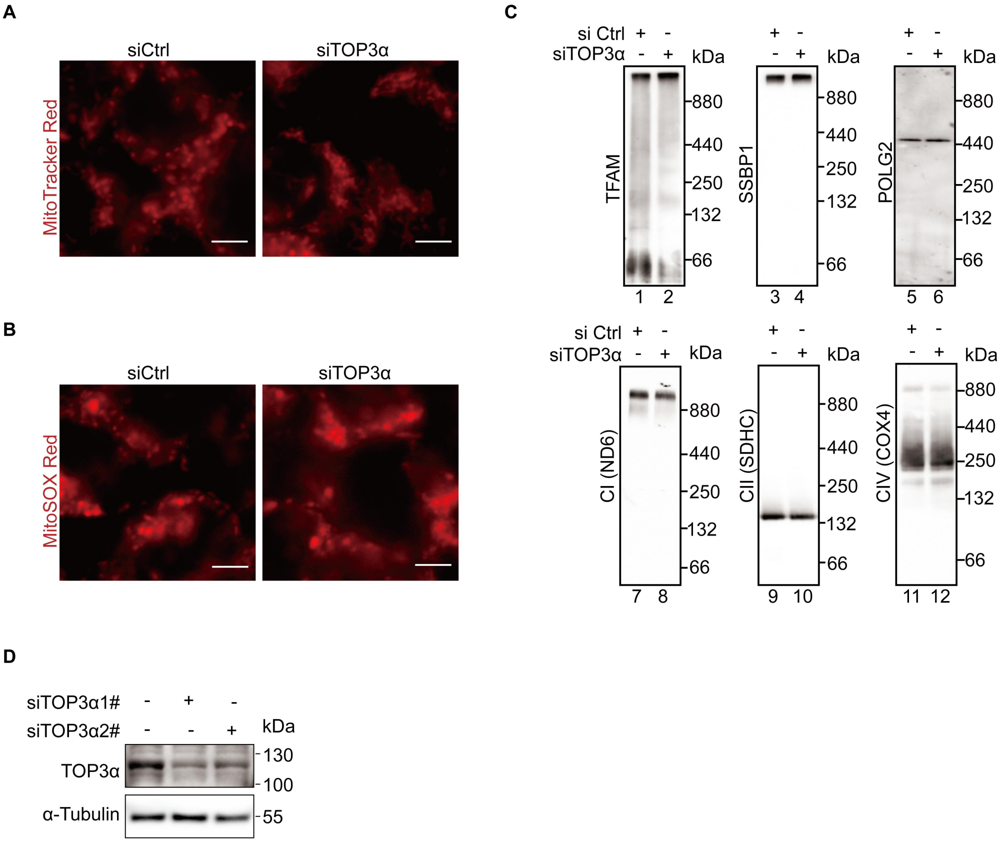

Appendix Figure S1. TOP3α downregulation does not alter mitochondrial membrane potential and ROS level.

(A) HEK-293T cells were treated with TOP3α siRNA 2# for 4 days and stained with MitoTracker Red for live-cell imaging. Scale bar = 20 μm.

(B) HEK-293T cells were treated as in (A) and stained with MitoSOX Red for live-cell imaging. Scale bar = 20 μm.

(C) HEK-293T cells were treated as in (A) and lysed with 1% digitonin for BN-PAGE and immunoblotting with indicated antibodies.

(D) HEK-293T cells were treated with TOP3α siRNA for 4 days and analyzed by immunoblotting using the indicated antibodies.

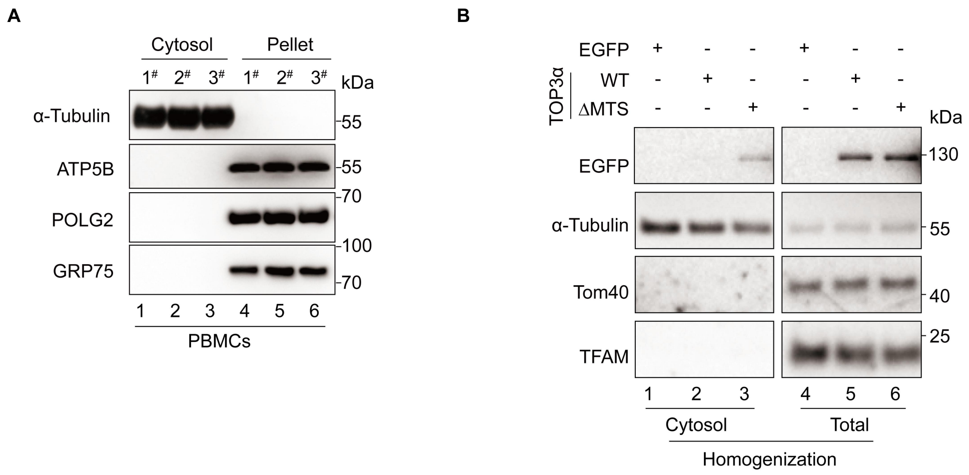

Appendix Figure S2. Subcellular fractionation does not cause the release of mitochondrial matrix components.

(A) Different PBMCs were subjected to 0.025% digitonin extraction followed by western blotting analysis of proteins from supernatant and pellet.

(B) HMC3 cells were transfected with plasmids expressing indicated proteins followed by homogenization and immunoblotting analysis.

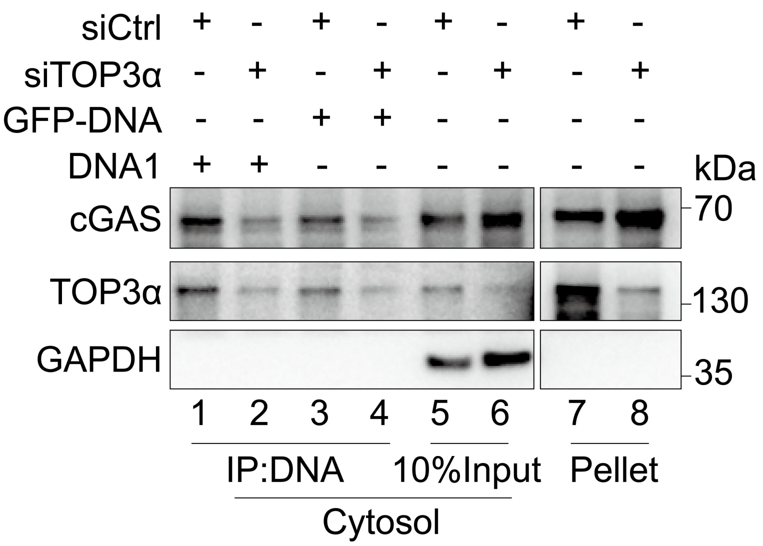

Appendix Figure S3. Cytosolic TOP3α promotes DNA-cGAS interaction.

HMC3 cells were transfected with TOP3α siRNA 2# for 4 days and subjected to interaction analysis with DNA. DNA1 (118 bp) was derived from 16S rRNA region of human mtDNA. GFP-DNA (118 bp) was amplified from EGFP cDNA with the GC content (50%) similar to that of DNA1 (47%).

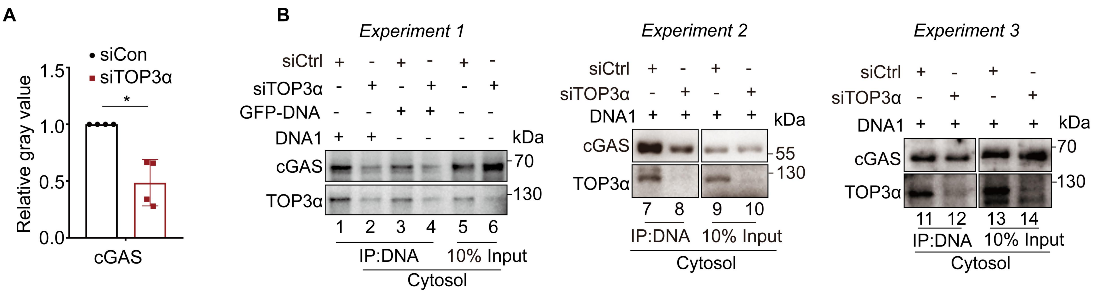

Appendix Figure S4. Depleting cytosolic TOP3α impairs mtDNA-cGAS interaction.

(A) Quantification of cGAS immunoprecipitated by DNA as in Figure 3C upon TOP3α depletion (n = 4 biologically independent experiments). Quantification was performed by ImageJ. The intensity of protein was subtracted by its corresponding background. The IP-enriched fraction was normalized to the corresponding input and then presented as ratio to the control group.

(B) Blots of experiments 1-3 used for quantification in (A). Experiment 1 was from Appendix Figure S3. Experiment 2 was from Figure 3C.

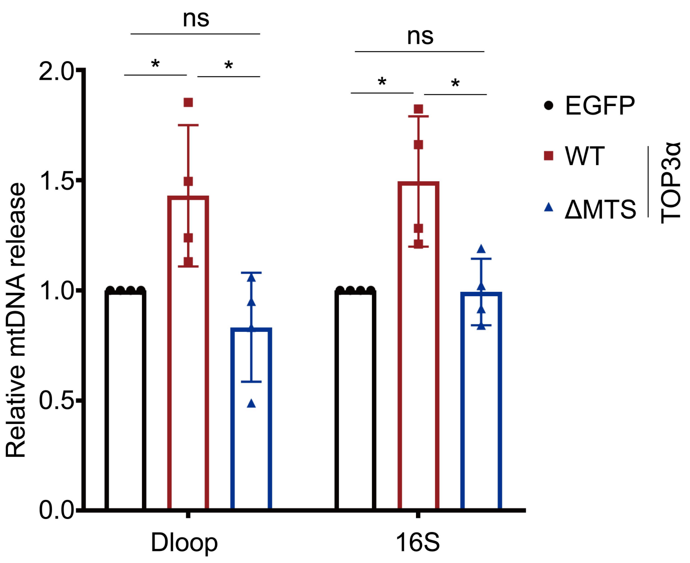

Appendix Figure S5. Mitochondrial overloaded TOP3α is required to cause mtDNA release.

Cytoplasmic fractions were isolated from HMC3 cells 3 days after TOP3α overexpression, and mtDNA release was measured by qPCR analysis. n = 4 biologically independent experiments.

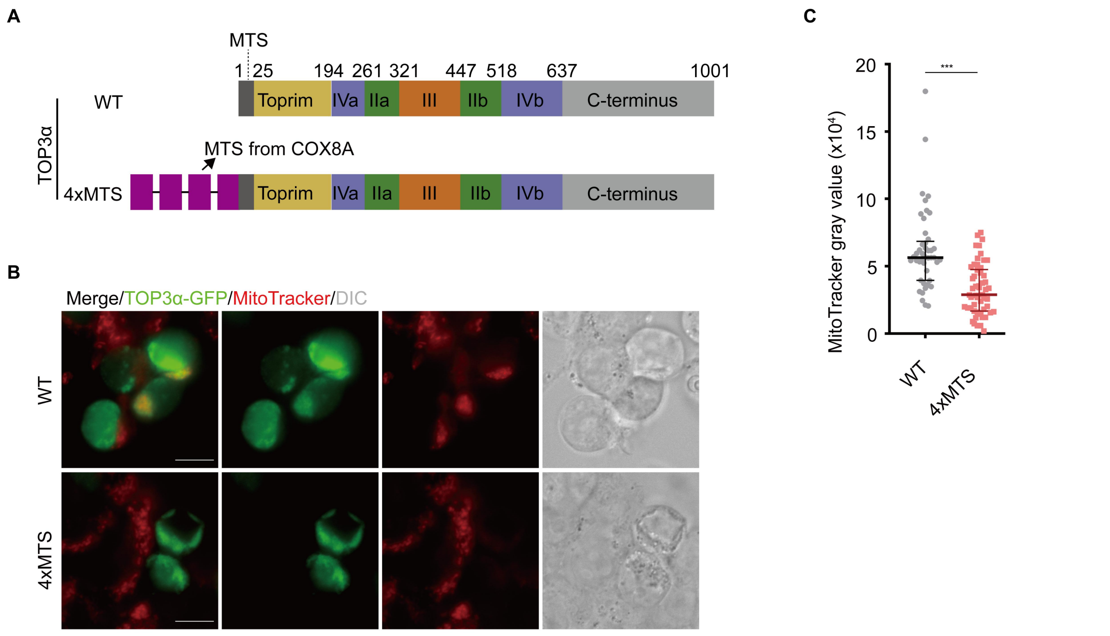

Appendix Figure S6. Enhancing mitochondrial targeting of TOP3α impairs mitochondrial membrane potential.

(A) Scheme of TOP3α constructs.

(B) HEK-293T cells were transfected with indicated constructs followed by MitoTracker Red staining and live-cell imaging. Scale bar = 20 μm.

(C) Quantification of mitochondrial membrane potential in (B). n ≥ 60 cells.

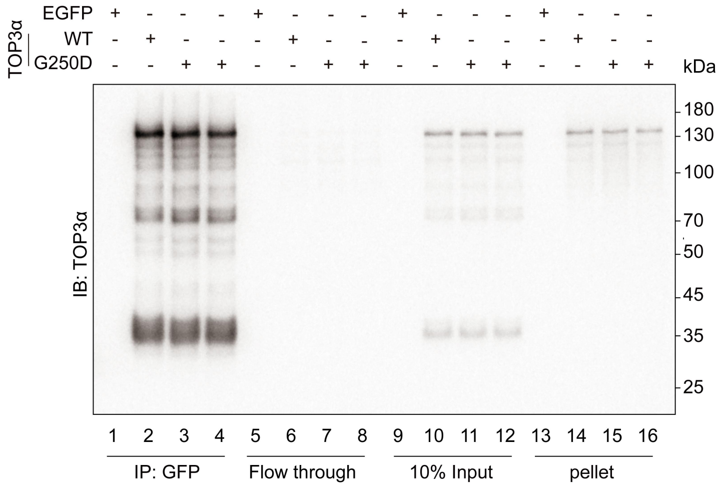

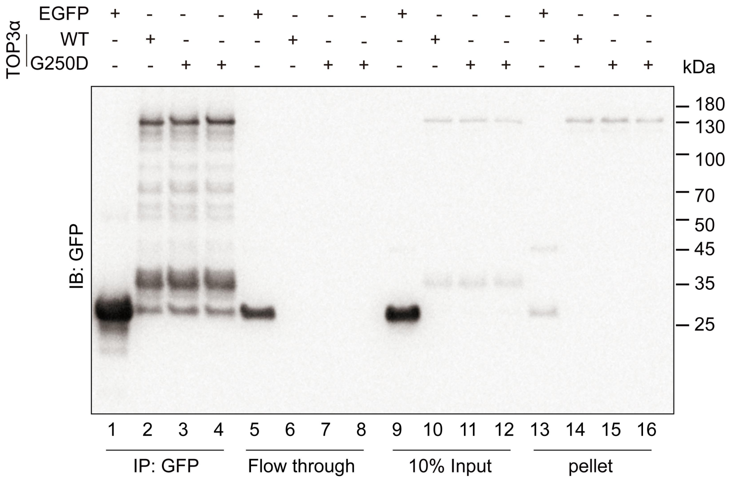

Appendix Figure S7. Complete blots of Figure EV4B.

The extraction of TOP3α is less efficient than TFAM (see Figure EV4A, the same sample) by 0.75% Trition X-100, possibly due to the nuclear presence of TOP3α. Note that the sample expressing G250D was loaded twice.

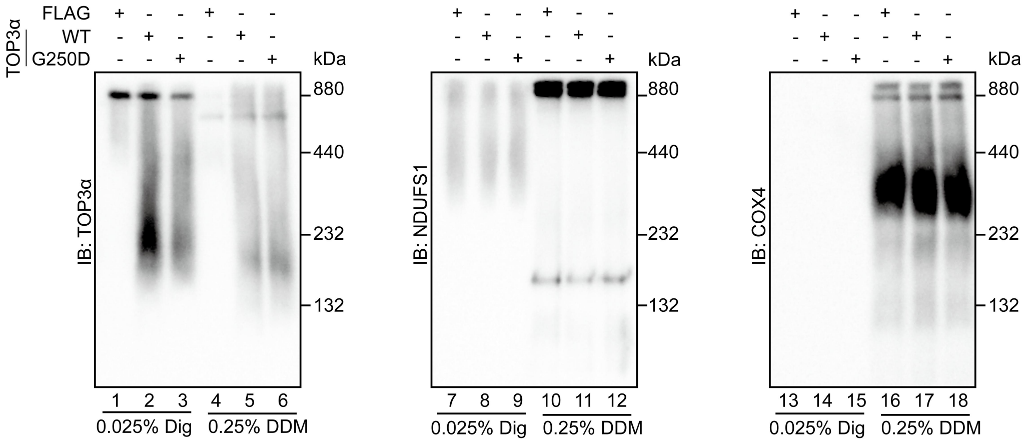

Appendix Figure S8. Loading control for Figure 5I.

Immunoblotting of cytosolic protein complexes extracted by 0.025% digitonin and pellet by 0.25% DDM of HMC3 cells. Lanes 1-3 detected by TOP3α antibodies were shown in Figure 5I.

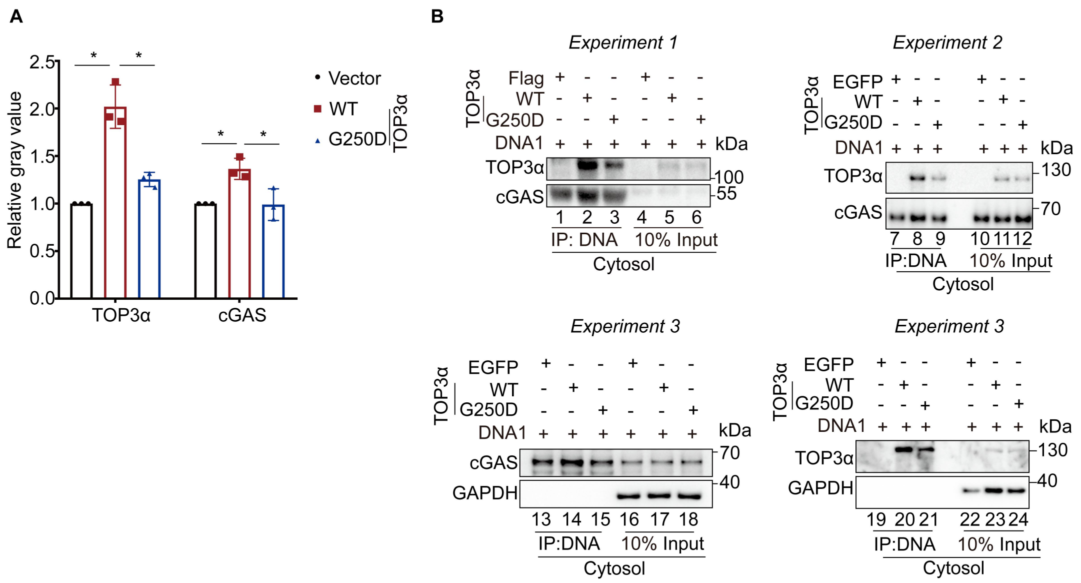

Appendix Figure S9. TOP3α-G250D suppresses the interaction of cGAS with mtDNA.

(A) Quantification of TOP3α and cGAS following DNA immunoprecipitation as in Figure 6B (n = 3 biologically independent experiments). Quantification was performed by ImageJ. The intensity of protein was subtracted by its corresponding background. The IP-enriched fraction was normalized to the corresponding input and then presented as ratio to the vector group.

(B) Blots of experiments 1-3 used for quantification in (A). Experiment 1 was from Figure EV4G. Experiment 2 was from Figure 6B. The sample of experiment 3 was run twice, because in the first analysis, the membrane of TOP3α was dirty.

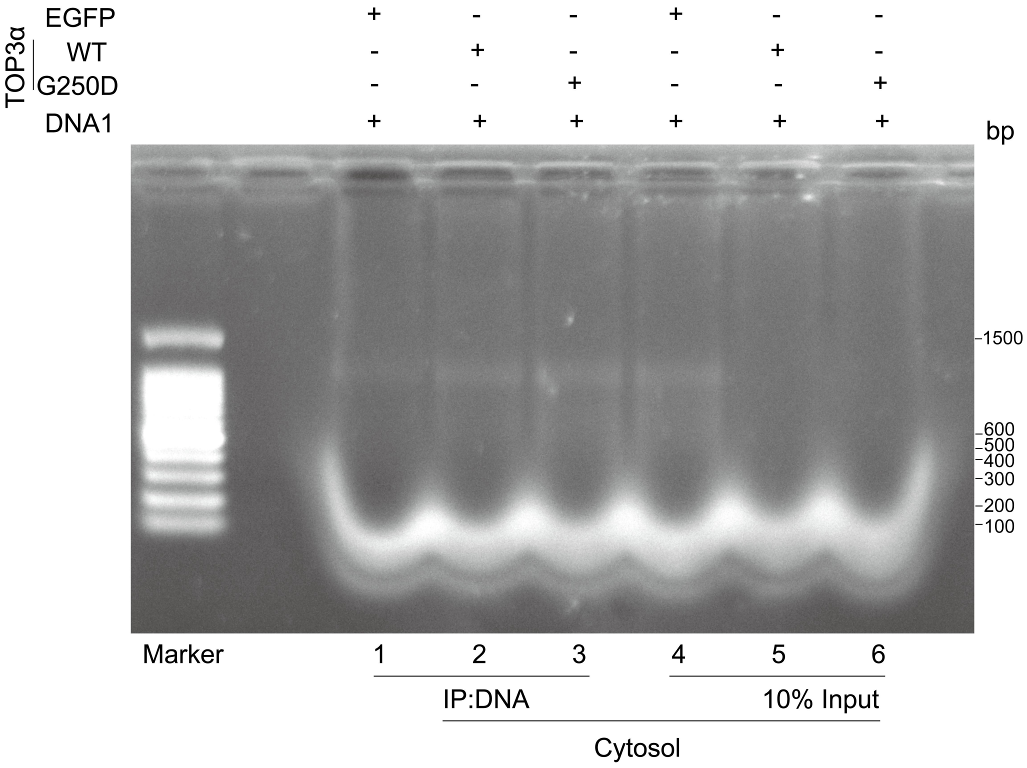

Appendix Figure S10. The IP efficiency of biotinylated DNA.

HMC3 cells expressing EGFP or EGFP tagged TOP3α constructs were subjected to cytosolic extraction using 0.025% digitonin followed by DNA interaction analysis. Streptavidin beads were boiled with SDS, and DNA was analyzed by 1% agarose gel stained by GelRed.

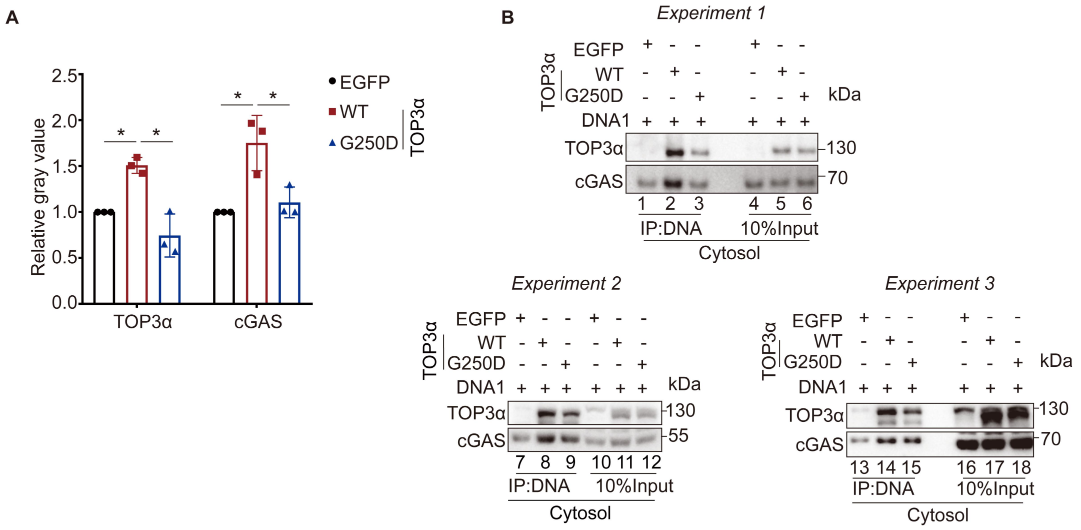

Appendix Figure S11. TOP3α-G250D suppresses the interaction of cGAS with mtDNA.

(A) Quantification of TOP3α and cGAS following DNA immunoprecipitation as in Figure 6D (n = 3 biologically independent experiments). Quantification was performed by ImageJ. The intensity of protein was subtracted by its corresponding background. The IP-enriched fraction was normalized to the corresponding input and then presented as ratio to the EGFP group.

(B) Blots of experiments 1-3 used for quantification in (A). Experiment 1 was from Figure 6D. Experiment 2 was from Figure EV4H.

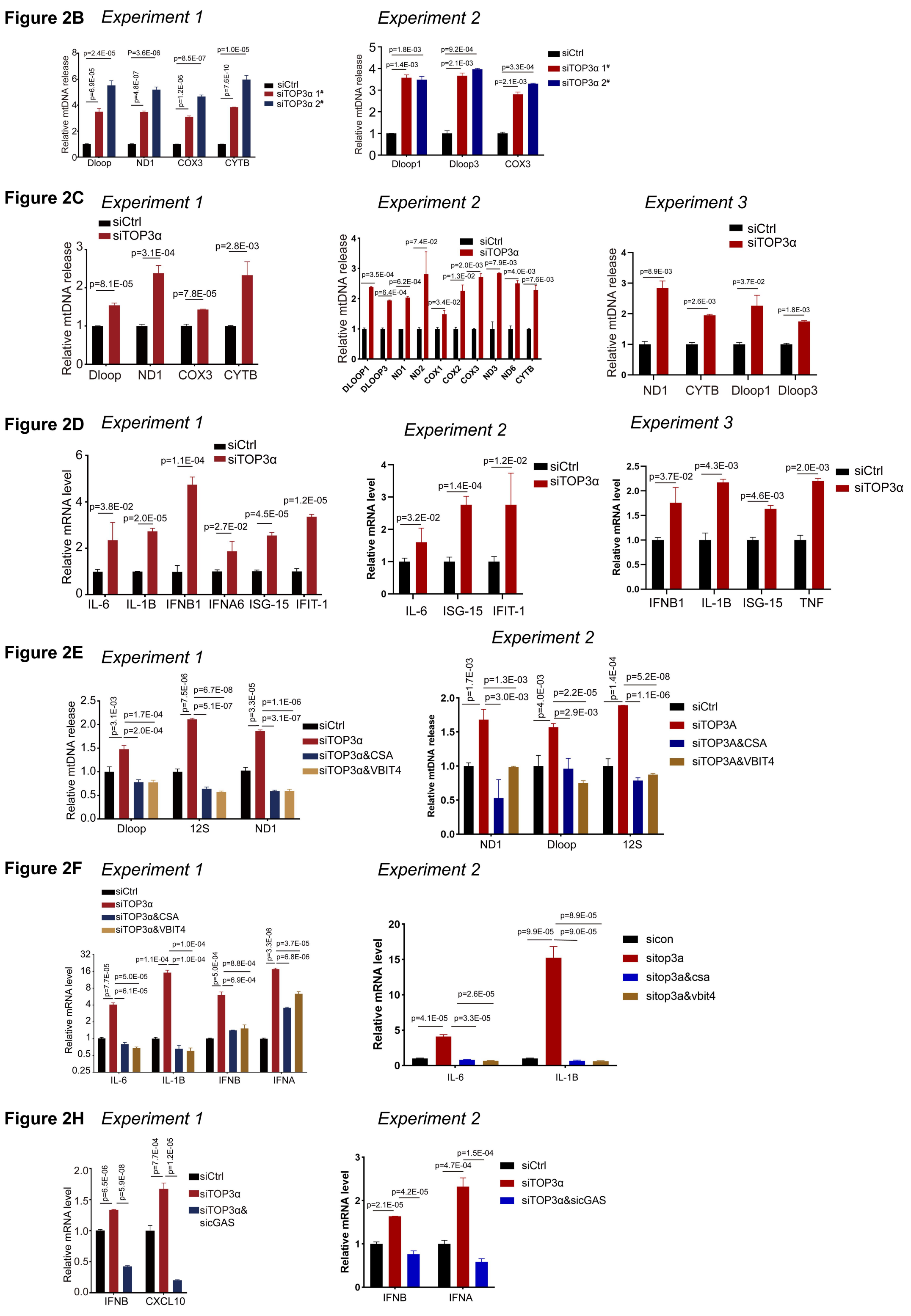

Appendix Figure S12. Independent repeat experiments related to Figure 2.

Figure 3G

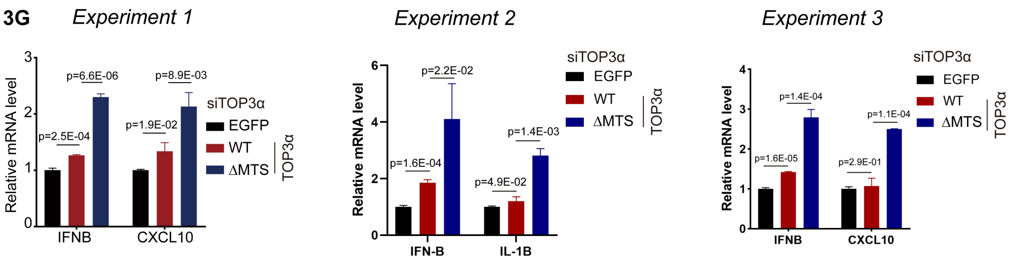

Figure 3I

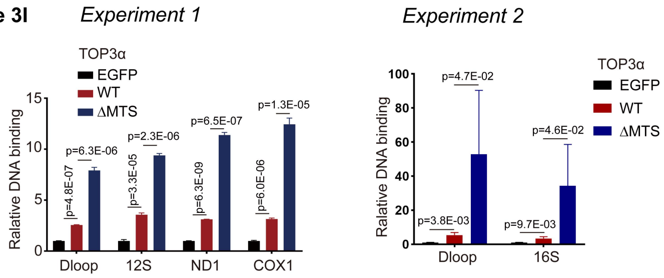

Figure 3K

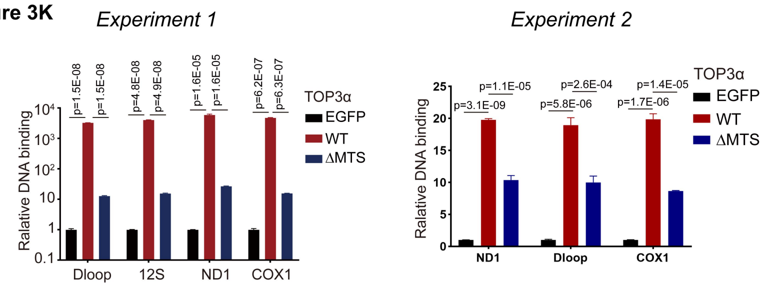

Appendix Figure S13. Independent repeat experiments related to Figure 3.

Figure 5F *Experiment 1* *Experiment 2*

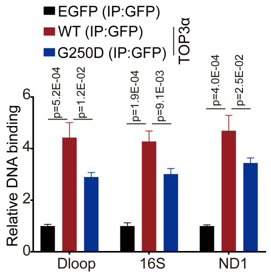

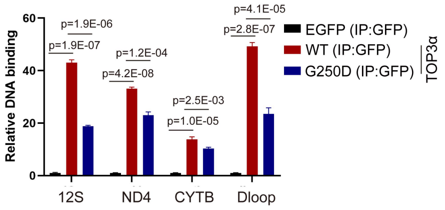

Figure 5H *Experiment 1* *Experiment 2*

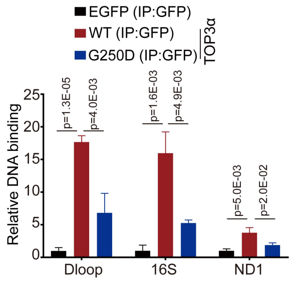

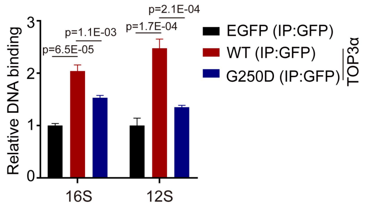

Appendix Figure S14. Independent repeat experiments related to Figure 5.

Figure 6F

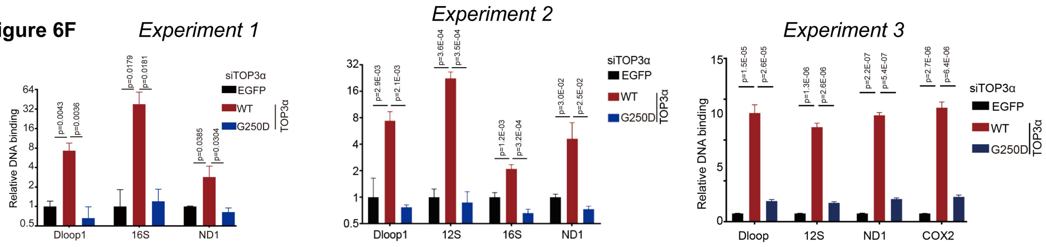

Figure 6G

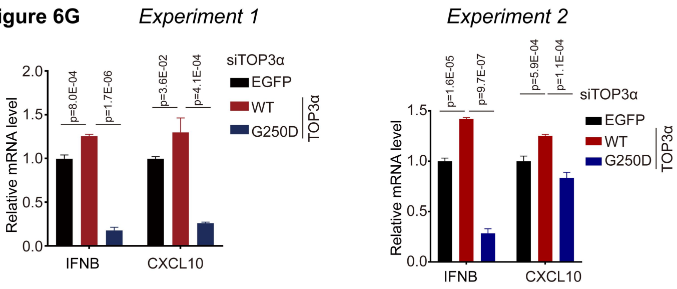

Figure 6H

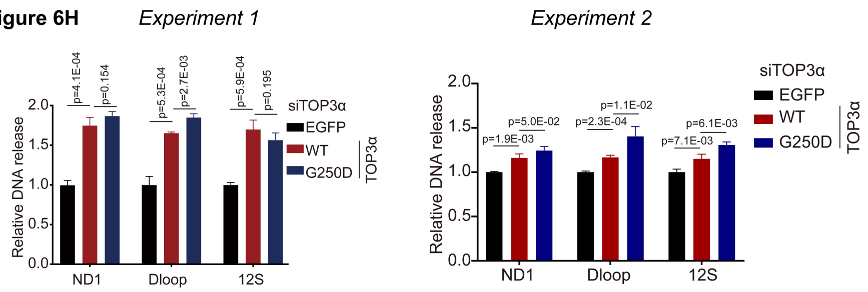

Appendix Figure S15. Independent repeat experiments related to Figure 6.

Figure EV3D *Experiment 1* *Experiment 2*

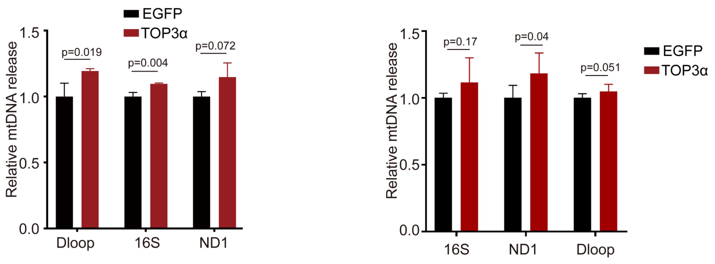

Figure EV3E *Experiment 1* *Experiment 2*

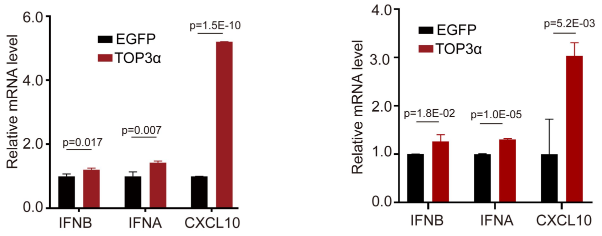

Appendix Figure S16. Independent repeat experiments related to Figure EV3.

Figure EV4C *Experiment 1* *Experiment 2*

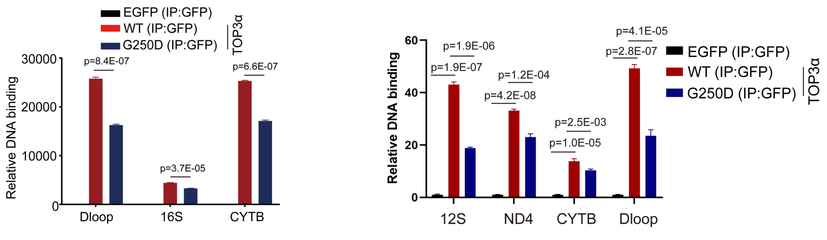

Figure EV4F *Experiment 1* *Experiment 2*

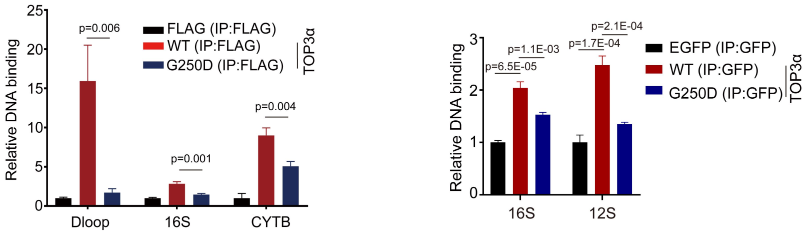

Appendix Figure S17. Independent repeat experiments related to Figure EV4.

Figure EV5B

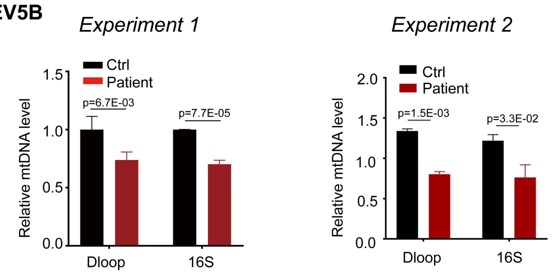

Figure EV5C

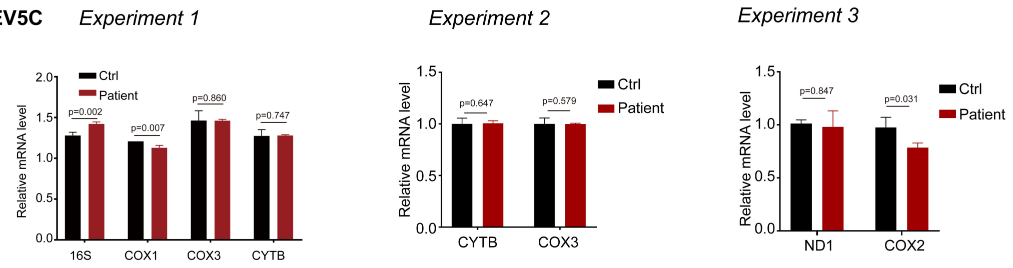

Figure EV5G

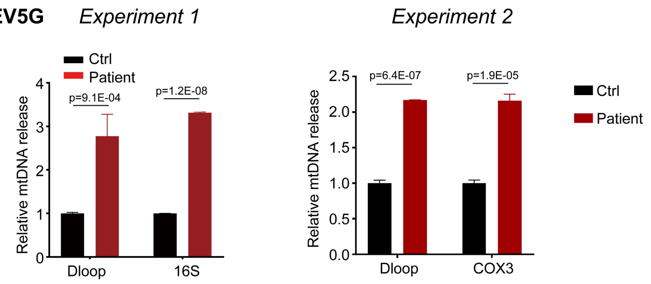

Figure EV5H

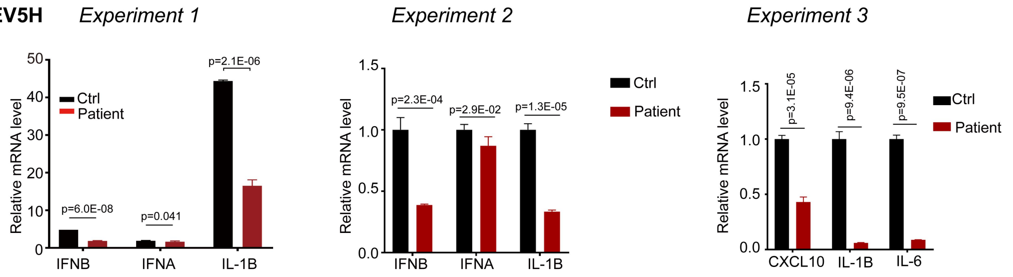

Figure EV5K

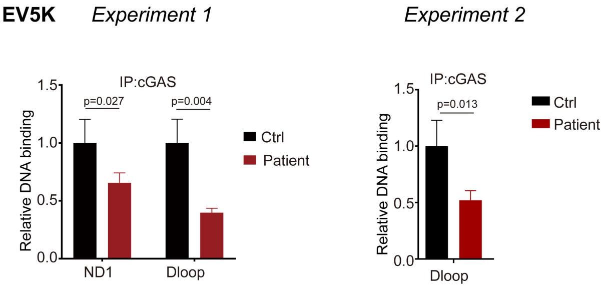

Appendix Figure S18. Independent repeat experiments related to Figure EV5.
