# Supplementary figures and images for: Cytosolic TOP3α facilitates mitochondrial DNA sensing by cGAS
Source: EMBO Rep. 2025 Oct 30;26(23):5959–81. doi: 10.1038/s44319-025-00614-2 (PMC12678531; doi:10.1038/s44319-025-00614-2)

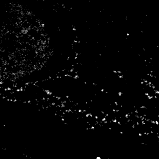

Supplement: Supplementary file 3 — Source data Fig. 1 [file 44319_2025_614_MOESM3_ESM.zip › Figure1/Figure1B/siCtrl.tif]

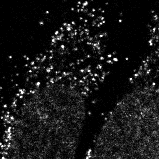

Supplement: Supplementary file 3 — Source data Fig. 1 [file 44319_2025_614_MOESM3_ESM.zip › Figure1/Figure1B/siTOP3α.tif]

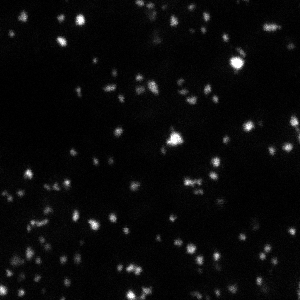

Supplement: Supplementary file 3 — Source data Fig. 1 [file 44319_2025_614_MOESM3_ESM.zip › Figure1/Figure1C/siCtrl.tif]

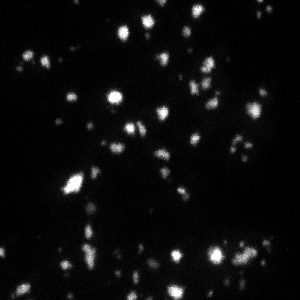

Supplement: Supplementary file 3 — Source data Fig. 1 [file 44319_2025_614_MOESM3_ESM.zip › Figure1/Figure1C/siTOP3α.tif]

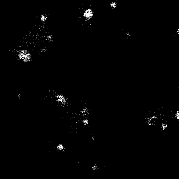

Supplement: Supplementary file 3 — Source data Fig. 1 [file 44319_2025_614_MOESM3_ESM.zip › Figure1/Figure1F/siCtrl.tif]

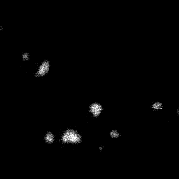

Supplement: Supplementary file 3 — Source data Fig. 1 [file 44319_2025_614_MOESM3_ESM.zip › Figure1/Figure1F/siTOP3α.tif]

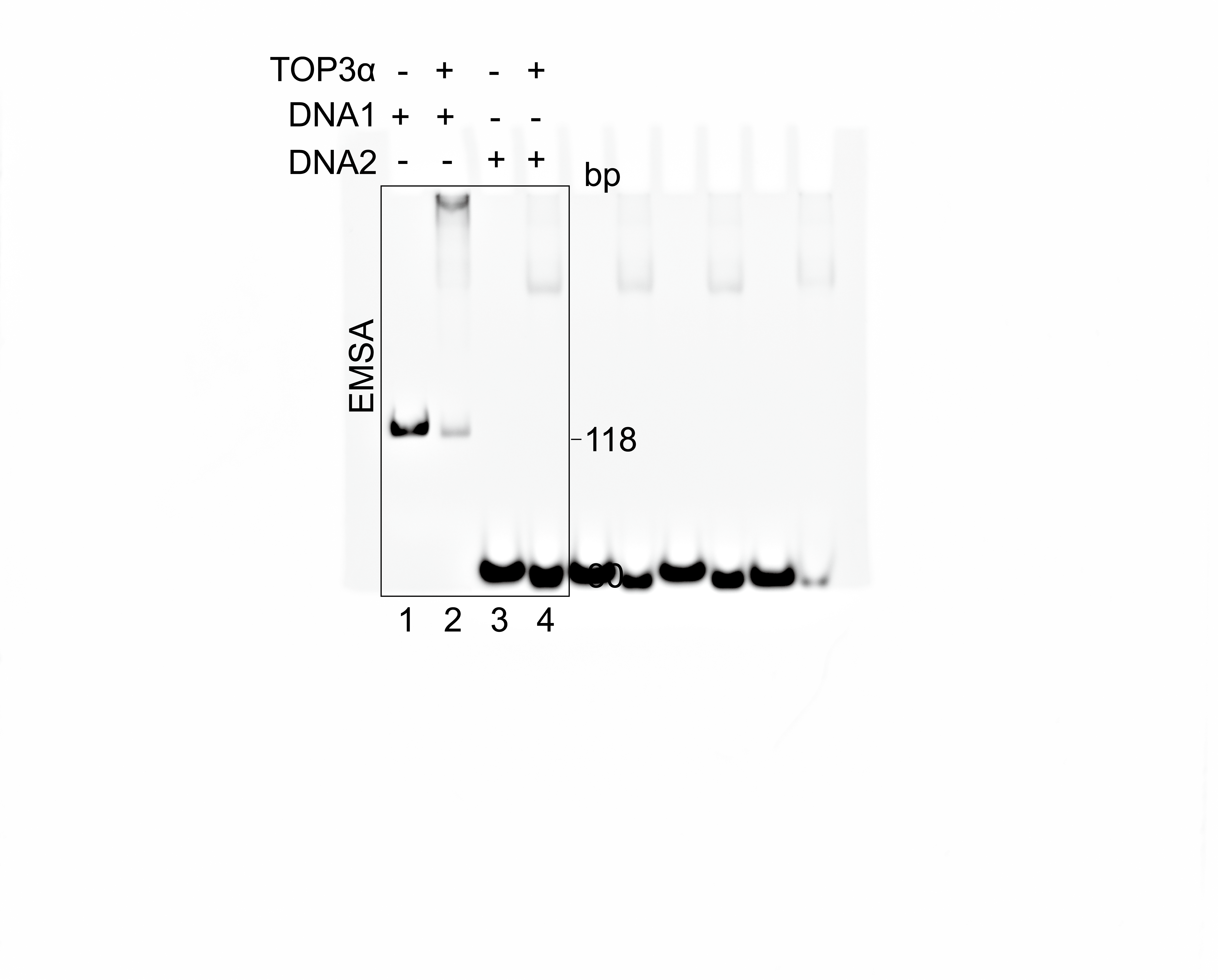

Supplement: Supplementary file 6 — Source data Fig. 4 [file 44319_2025_614_MOESM6_ESM.zip › Figure4/Figure4D/Figure4D.tif]
