# Supplementary material for: Cytosolic TOP3α facilitates mitochondrial DNA sensing by cGAS
Source: EMBO Rep. 2025 Oct 30;26(23):5959–81. doi: 10.1038/s44319-025-00614-2 (PMC12678531; doi:10.1038/s44319-025-00614-2)
Supplement: Supplementary file 9 — Expanded View Figures [file 44319_2025_614_MOESM9_ESM.pdf]

Expanded View Figures

Figure EV1. Knocking-down TOP3α causes mtDNA clustering and release.

(**A**) TOP3α level was knocked down by siRNA for 4 days in U2OS cells followed by immunofluorescence. Scale bar = 20 μm. (**B**) Immunofluorescence of U2OS cells following treatment as in (**A**). White squared area was zoomed in. Images were captured by Apotome microscopy. Scale bar (overview) = 20 μm, scale bar (zoom) = 2 μm. (**C**) Immunofluorescence of U2OS cells following treatment as in (**A**). White squared area was zoomed in. The released mtDNA was marked by white arrowhead. Scale bar (overview) = 20 μm, scale bar (zoom) = 2 μm. (**D**) The number of released mtDNA was quantified in (**C**). Results were mean ± SD, $n = 7$. Unpaired t-test was used for statistical analysis. (**E**) The intensity of released mtDNA was quantified in (**C**). Results were mean ± SD, $n = 7$. Unpaired t-test was used for statistical analysis. (**F**) Immunofluorescence of U2OS cells following treatment as in (**A**). White squared area was zoomed in. The released mtDNA was marked by white arrowhead. Images were captured by HIS-SIM microscopy. Scale bar (overview) = 20 μm, scale bar (zoom) = 2 μm. (**G**) Plot of immunofluorescent intensity of DNA and Tom40 along the white line drawn in (**F**).

 © The Author(s)

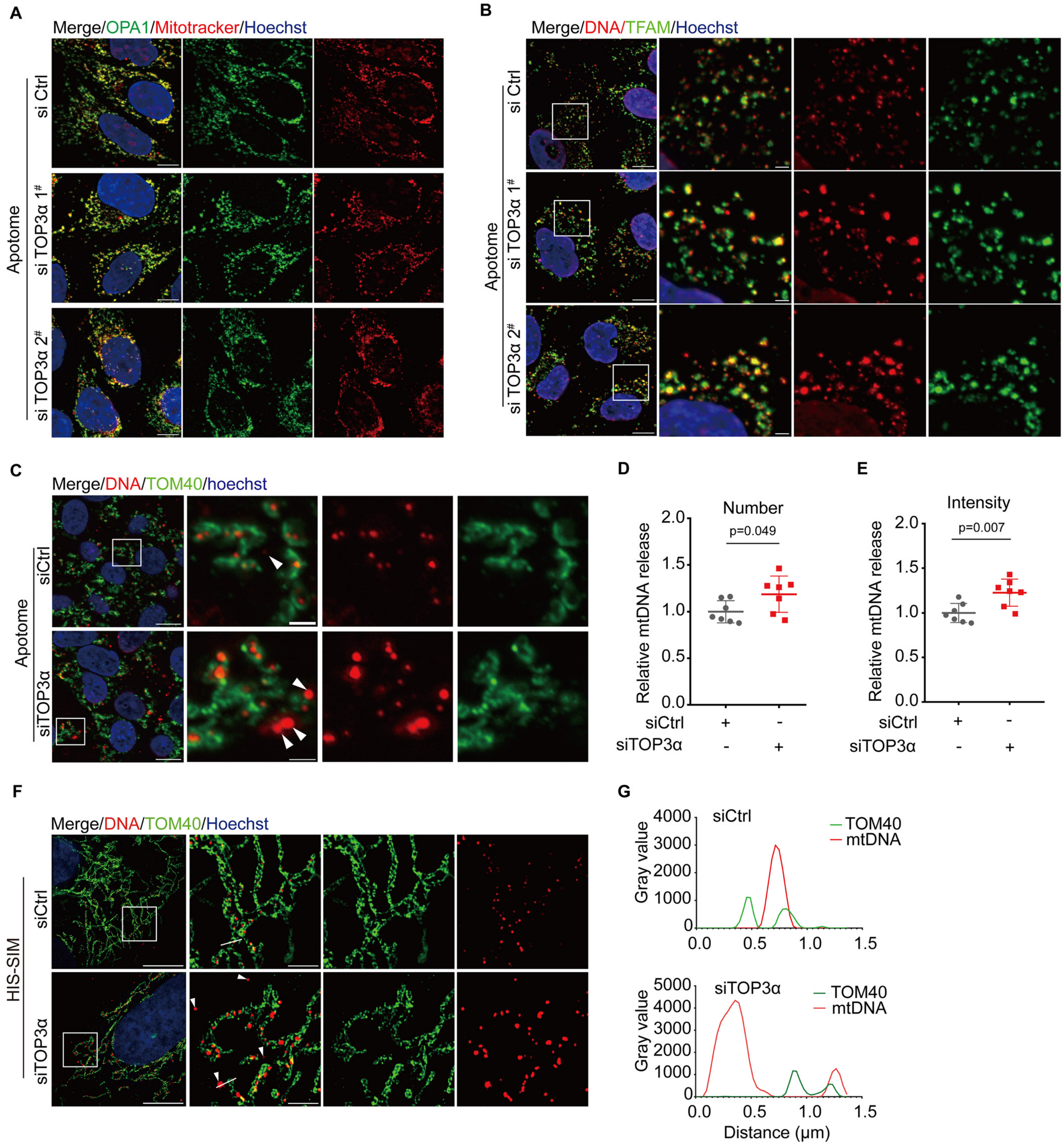
A
Merge/OPA1/Mitotracker/Hoechst
Apotome
si Ctrl
si TOP3α 1#
si TOP3α 2#
B
Merge/DNA/TFAM/Hoechst
Apotome
si Ctrl
si TOP3α 1#
si TOP3α 2#
C
Merge/DNA/TOM40/hoechst
Apotome
siCtrl
siTOP3α
D
Number
p=0.049
Relative mtDNA release
2.0
1.5
1.0
0.5
0.0
siCtrl + -
siTOP3α - +
E
Intensity
p=0.007
Relative mtDNA release
2.0
1.5
1.0
0.5
0.0
siCtrl + -
siTOP3α - +
F
Merge/DNA/TOM40/Hoechst
HIS-SIM
siCtrl
siTOP3α
G
siCtrl
TOM40
mtDNA
Gray value
4000
3000
2000
1000
0
0.0 0.5 1.0 1.5
siTOP3α
TOM40
mtDNA
Gray value
5000
4000
3000
2000
1000
0
0.0 0.5 1.0 1.5
Distance (μm)

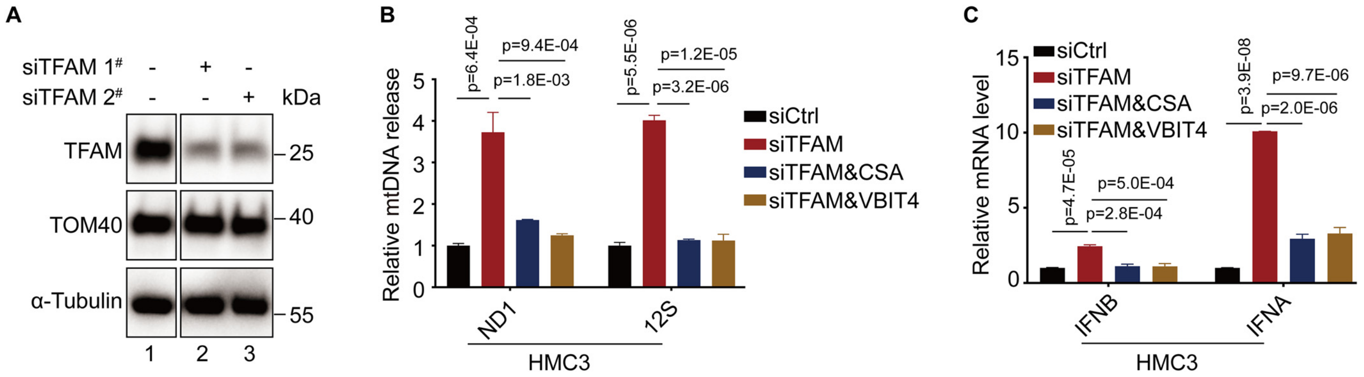

Figure EV2. Knocking-down TFAM induces mtDNA release and inflammation.

(**A**) Depletion of TFAM by siRNA followed by immunoblotting with indicated antibodies. (**B**) TFAM was knocked down by siRNA in HMC3 cells for 4 days and treated with CsA or VBIT-4 for the last 2 days followed by qPCR analysis for released mtDNA. Results were mean ± SD, $n = 3$ technical replicates. Unpaired *t*-test was used for statistical analysis. (**C**) HMC3 cells were treated as in (**B**) and subjected to qPCR assay for mRNA level (normalized to ACTB). Results were mean ± SD, $n = 3$ technical replicates. Unpaired *t*-test was used for statistical analysis.

 © The Author(s)

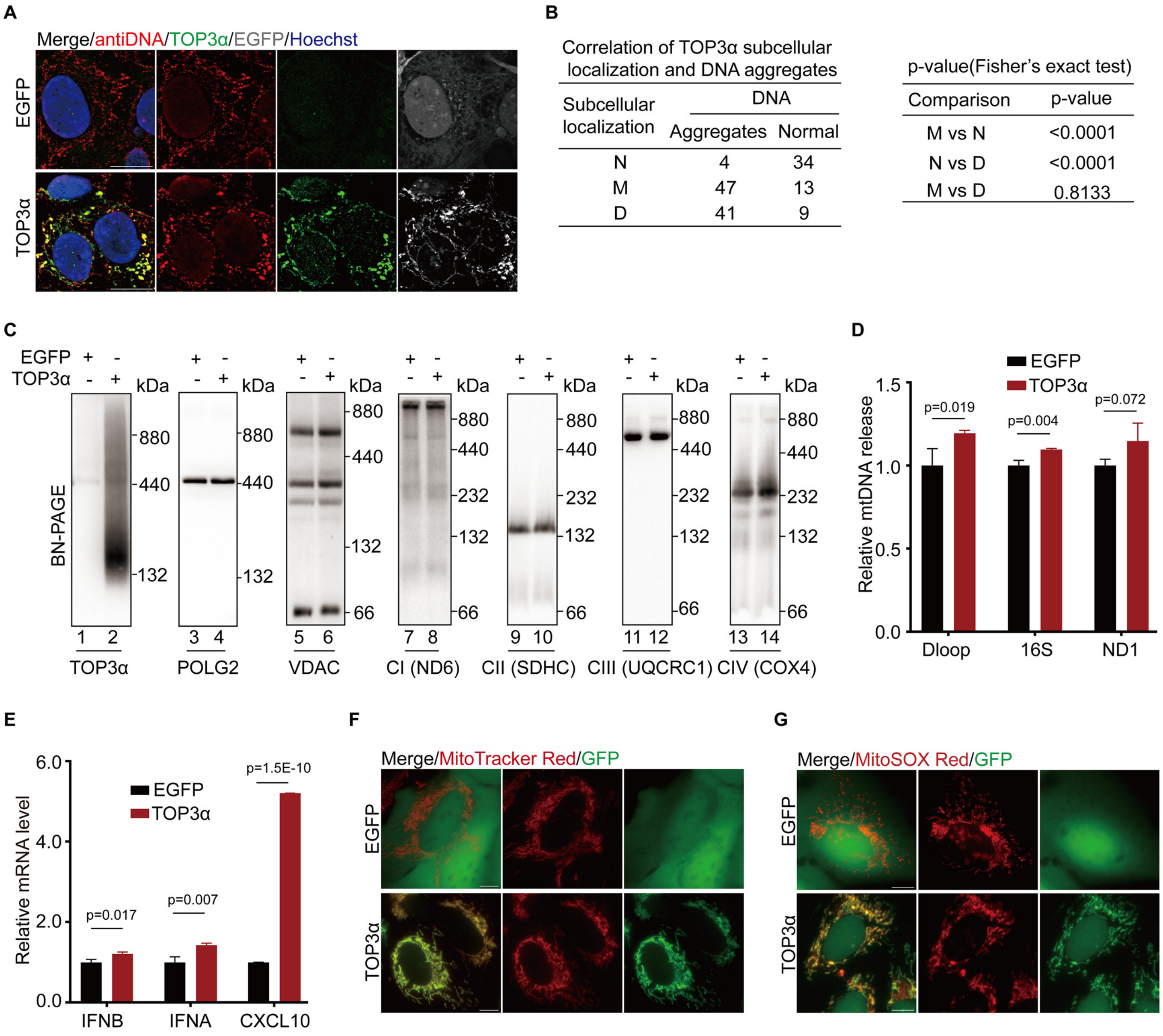

Figure EV3. TOP3α overexpression leads to mtDNA aggregation and inflammatory response.

(**A**) Immunofluorescence of U2OS cells expressing EGFP or EGFP tagged TOP3α for 3 days, scale bar = 20 μm. (**B**) Correlation of TOP3α subcellular localization and mtDNA aggregation. N: nucleus; M: mitochondria; D: dual localization. Fisher's exact test was used for statistical analysis. (**C**) Proteins of U2OS cells expressing EGFP or EGFP tagged TOP3α for 3 days were extracted by 1% digitonin and separated by BN-PAGE followed by immunoblotting with indicated antibodies. (**D**) U2OS cells expressing EGFP or EGFP tagged TOP3α for 3 days were analyzed by qPCR for cytosolic mtDNA with indicated primers. Results were mean ± SD, $n = 3$ technical replicates. Unpaired *t*-test was used for statistical analysis. (**E**) U2OS cells expressing EGFP or EGFP tagged TOP3α for 3 days were analyzed by qPCR for indicated mRNA (normalized to ACTB). Results were mean ± SD, $n = 3$ technical replicates. Unpaired *t*-test was used for statistical analysis. (**F**) U2OS cells expressing EGFP or EGFP tagged TOP3α for 3 days were stained with MitoTracker Red for live-cell imaging. Scale bar = 20 μm. (**G**) U2OS cells expressing EGFP or EGFP tagged TOP3α for 3 days were stained with MitoSOX Red for live-cell imaging. Scale bar = 20 μm.

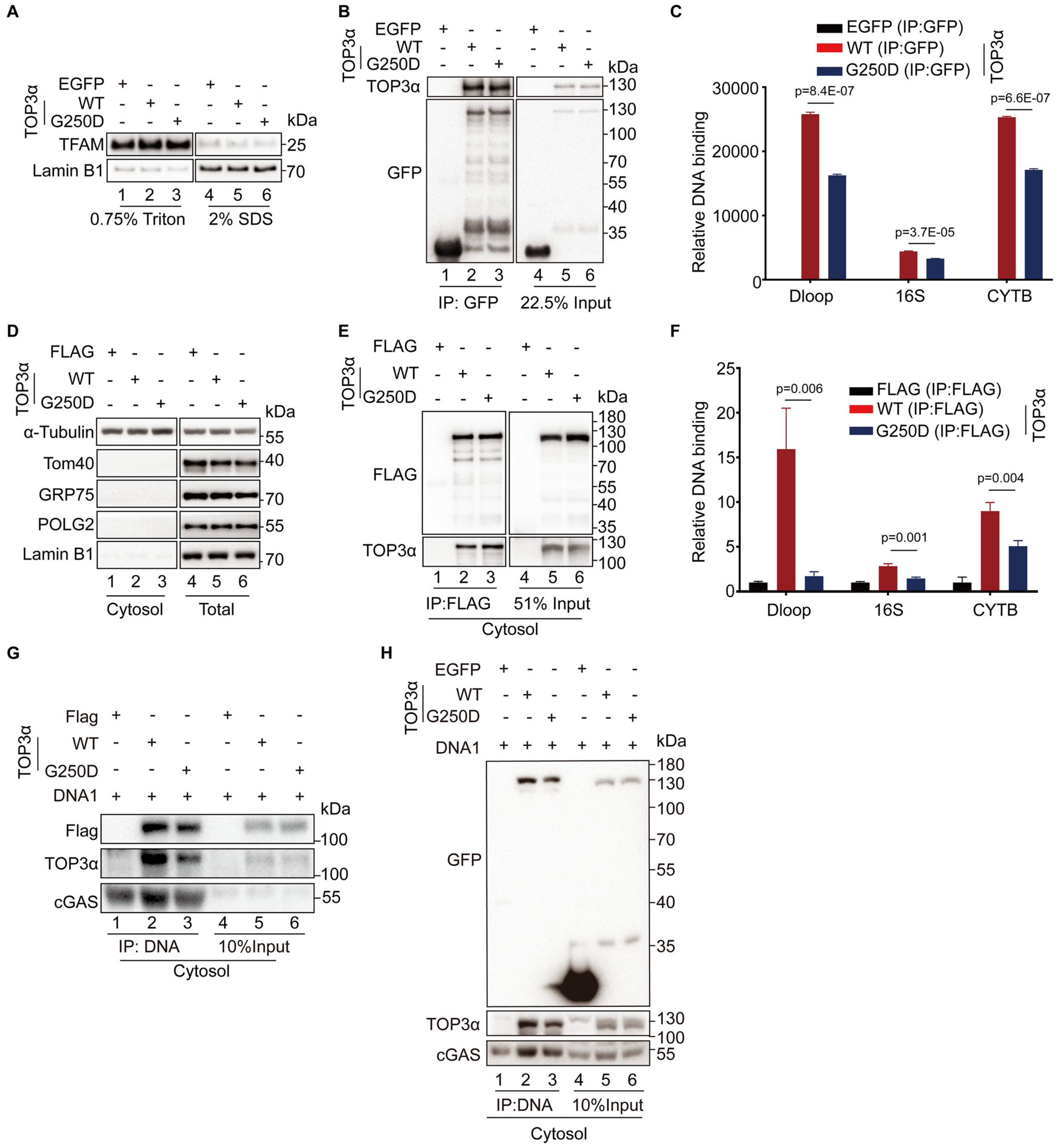
A
EGFP
WT
G250D
TOP3α
TFAM
Lamin B1
kDa
25
70
1 2 3 4 5 6
0.75% Triton
2% SDS
B
EGFP
WT
G250D
TOP3α
GFP
kDa
130
100
70
55
40
35
1 2 3 4 5 6
IP: GFP
22.5% Input
C
EGFP (IP:GFP)
WT (IP:GFP)
G250D (IP:GFP)
Relative DNA binding
30000
20000
10000
0
p=8.4E-07
p=3.7E-05
p=6.6E-07
Dloop
16S
CYTB
D
FLAG
WT
G250D
α-Tubulin
Tom40
GRP75
POLG2
Lamin B1
kDa
55
40
70
1 2 3 4 5 6
Cytosol
Total
E
FLAG
WT
G250D
kDa
180
130
100
70
55
40
35
TOP3α
IP:FLAG
51% Input
Cytosol
F
25
20
15
10
5
0
p=0.006
p=0.001
p=0.004
FLAG (IP:FLAG)
WT (IP:FLAG)
G250D (IP:FLAG)
G
Flag
WT
G250D
DNA1
TOP3α
cGAS
IP: DNA
10%Input
Cytosol
H
EGFP
IP:DNA
10%Input

Figure EV4. G250D mutation impairs the binding of TOP3α and cGAS to mtDNA.

(**A**) Immunoblotting of U2OS cells expressing EGFP or EGFP tagged TOP3α for 2 days. Proteins were extracted by 0.75% Triton X-100 (supernatant) and proteins left in pellet were extracted by 2% SDS. (**B**) Co-immunoprecipitation of U2OS cells following treatment as in (**A**). TOP3α proteins extracted by 0.75% Triton X-100 were pulled down by anti-GFP beads. (**C**) qPCR analysis of mtDNA bound to TOP3α following co-immunoprecipitation as in (**B**). Results were mean ± SD, $n = 3$ technical replicates. Unpaired *t*-test was used for statistical analysis. (**D**) Immunoblotting of U2OS cells following transfection of empty vector or corresponding plasmids encoding FLAG-tagged TOP3α constructs for 2 days. Cytosolic fraction was extracted by 0.025% digitonin and the whole-cell lysate was extracted by 2% SDS. (**E**) Co-immunoprecipitation of U2OS cells following treatment as in (**D**). Cytosolic TOP3α proteins extracted by 0.025% digitonin were pulled down by anti-FLAG beads. (**F**) qPCR analysis of mtDNA bound to cytosolic TOP3α following co-immunoprecipitation as in (**E**). Results were mean ± SD, $n = 3$ technical replicates. Unpaired *t*-test was used for statistical analysis. (**G**) The biotin-labeled DNA was transfected into U2OS cells for pull down of cytosolic components by streptavidin beads as in Fig. 6A. U2OS cells were transfected with empty vector or corresponding plasmids encoding FLAG-tagged TOP3α constructs for 2 days. (**H**) U2OS cells expressing EGFP or EGFP tagged TOP3α for 2 days and subjected to interaction analysis as in Fig. 6C.

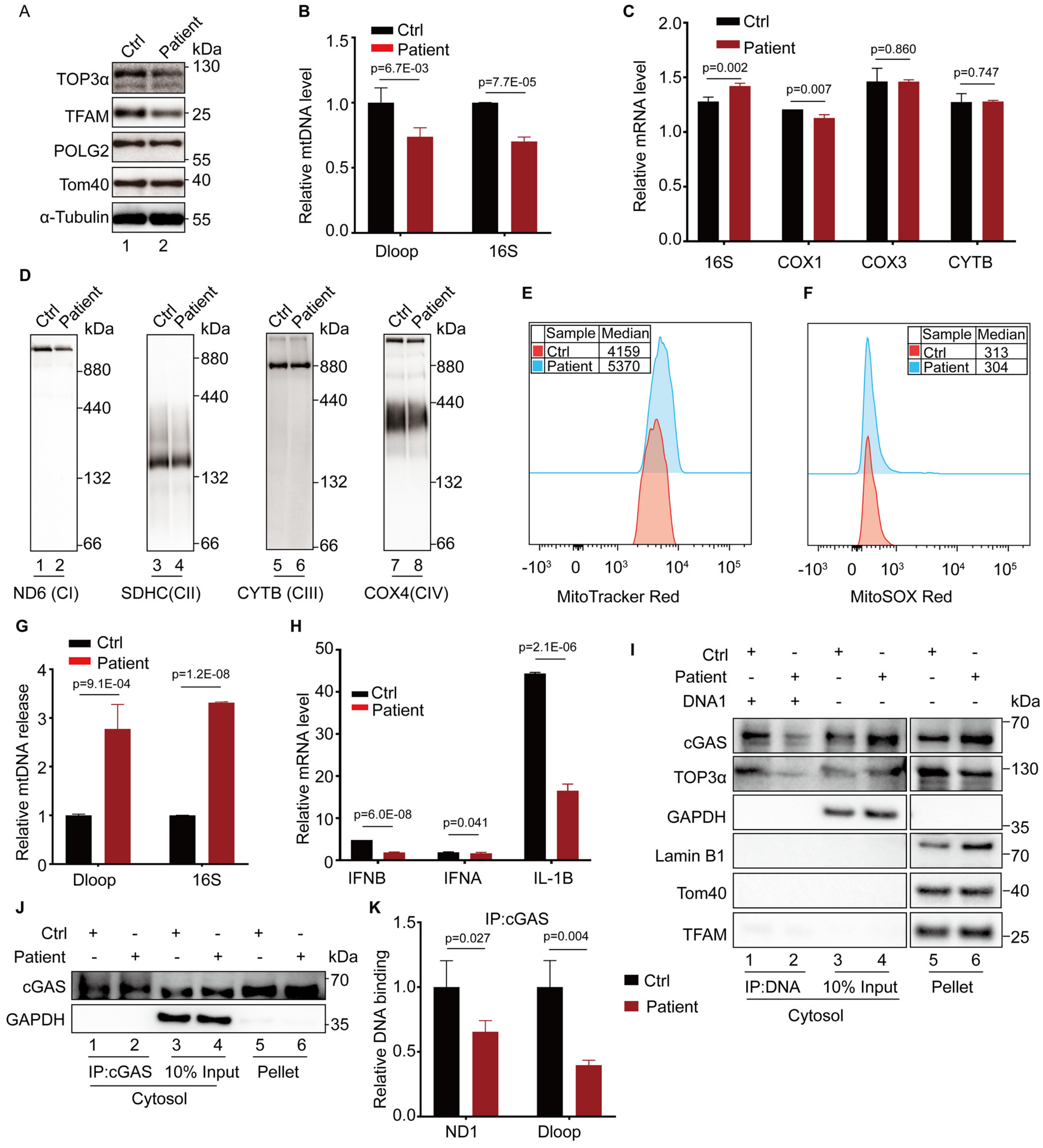
A
Ctrl
Patient
kDa
TOP3α
TFAM
POLG2
Tom40
α-Tubulin
130
25
55
40
55
1 2
B
Ctrl
Patient
p=6.7E-03
p=7.7E-05
Relative mtDNA level
Dloop
16S
C
Ctrl
Patient
p=0.002
p=0.007
p=0.860
p=0.747
Relative mRNA level
16S
COX1
COX3
CYTB
D
Ctrl
Patient
kDa
880
440
132
66
1 2
3 4
5 6
7 8
ND6 (CI)
SDHC(CII)
CYTB (CIII)
COX4(CIV)
E
Sample Median
Ctrl 4159
Patient 5370
MitoTracker Red
F
Sample Median
Ctrl 313
Patient 304
MitoSOX Red
G
Ctrl
Patient
p=9.1E-04
p=1.2E-08
Relative mtDNA release
Dloop
16S
H
Ctrl
Patient
p=6.0E-08
p=0.041
p=2.1E-06
Relative mRNA level
IFNB
IFNA
IL-1B
I
Ctrl
Patient
DNA1
kDa
cGAS
TOP3α
GAPDH
Lamin B1
Tom40
TFAM
70
130
35
70
40
25
1 2 3 4 5 6
IP:DNA
10% Input
Pellet
Cytosol
J
Ctrl
Patient
kDa
cGAS
GAPDH
70
35
1 2 3 4 5 6
IP:cGAS
10% Input
Pellet
Cytosol
K
IP:cGAS
p=0.027
p=0.004
Relative DNA binding
ND1
Dloop
Ctrl
Patient

 © The Author(s)

Figure EV5. G250D mutation impairs mtDNA interaction with cytosolic TOP3α and cGAS in patient-derived PBMCs.

(**A**) Immunoblotting of peripheral blood mononuclear cells (PBMCs) isolated from ALS patient and a control in this family. (**B**) qPCR for mtDNA copy number in PBMCs. Results were mean ± SD, $n = 3$ technical replicates. Unpaired *t*-test was used for statistical analysis. (**C**) qPCR for mRNA level in PBMCs. Results were mean ± SD, $n = 3$ technical replicates. Unpaired *t*-test was used for statistical analysis. (**D**) Immunoblotting of protein complexes extracted by 1% digitonin of PBMCs. (**E**) Flow cytometry of PBMCs, cells were stained with 100 nM MitoTracker Red for 30 min. (**F**) Flow cytometry of PBMCs, cells were stained with 5 μM MitoSOX Red for 30 min. (**G**) qPCR for mtDNA release in PBMCs. Results were mean ± SD, $n = 3$ technical replicates. Unpaired *t*-test was used for statistical analysis. (**H**) qPCR for mRNA level in PBMCs. Results were mean ± SD, $n = 3$ technical replicates. Unpaired *t*-test was used for statistical analysis. (**I**) Cytosolic components of PBMCs were extracted using 0.025% digitonin and subjected to immunoprecipitation by biotin-labeled DNA. The pellets were lysed with 2% SDS. (**J**) Co-immunoprecipitation of cytosolic components via anti-cGAS beads. Cytosolic and pellet components were extracted as described in (**I**). (**K**) qPCR analysis of mtDNA bound to endogenous cGAS following co-immunoprecipitation as in (**J**). Results were mean ± SD, $n = 3$ technical replicates. Unpaired *t*-test was used for statistical analysis.

© The Author(s)
